# IL-34 promotes foam cell formation by enhancing CD36 expression through p38 MAPK pathway

**DOI:** 10.1038/s41598-018-35485-2

**Published:** 2018-11-26

**Authors:** Qingyan Liu, Jiao Fan, Jing Bai, Liang Peng, Tao Zhang, Lei Deng, Gaokun Wang, Yu Zhao, Jingguo Nong, Minghua Zhang, Yu Wang

**Affiliations:** 1 Comprehensive Liver Cancer Center, 302 Military Hospital of China, Beijing, 100039 China; 2 Institute of Geriatrics, National Clinical Research Center of Geriatrics Disease, Chinese PLA General Hospital, Beijing, 100853 China; 3 Department of Cardiology, Chinese PLA General Hospital, Beijing, 100853 China; 4 Department of Cardiology, Henan Provincial People’s Hospital, Zhengzhou, 450003 China; 5 Clinical Pharmacy Laboratory, Chinese PLA General Hospital, Beijing, 100853 China

## Abstract

Atherosclerosis is characterized as a chronic inflammatory disease and macrophage-derived foam cells play a central role during the pathologic processes. A newly discovered cytokine interleukin-34 (IL-34) is closely associated with various inflammatory and autoimmune diseases. Expression of IL-34 in obesity, inflammatory bowel disease (IBD), rheumatoid arthritis (RA), lupus nephritis and coronary artery diseases (CAD) are significantly elevated. However, the role of IL-34 in atherosclerosis remains unknown. In our present study, we found that IL-34 treatment markedly increased the uptake of oxLDL, intracellular total and esterified cholesterol content but not cholesterol efflux, subsequently promoted foam cell formation through up-regulating CD36 expression via p38 MAPK signal pathway in bone marrow derived macrophages (BMDMs). Furthermore, treatment with IL-34 significantly elevated the oxLDL-induced up-regulation of pro-inflammatory cytokines. Our results conclude that IL-34 facilitates foam cell formation by increasing CD36-mediated lipid uptake and suggest a potential new risk biomarker for atherosclerosis.

## Introduction

Cardiovascular diseases still remain to be the leading cause of mortality and morbidity worldwide^1,2^. Atherosclerosis, a chronic progressive inflammatory disease, is the fundamental pathophysiological process underlying coronary artery disease. The rupture of atherosclerotic plaques and thrombosis can lead to life-threatening clinical complication including myocardial infarction, stroke or sudden death. Foam cell formation has been considered as a central pathologic progress responsible for the initial stage of atherosclerosis^3^. Macrophages infiltrate into the subendothelial space and intake modified low-density lipoproteins (LDL), such as oxidized-LDL (oxLDL) and acetylated-LDL (acLDL), leading to the accumulation of intra-cellular lipids and then become lipid-laden foam cells via scavenger receptors, including CD36, SR-A, and lectin-like oxLDL receptor-1 (Lox-1), key members of scavenger receptors family^4,5^. Cholesterol efflux is mediated by ATP-binding cassette transporters A1 and G1 (ABCA1 and ABCG1), and scavenger receptor class B type 1 (SR-B)^6–8^. The disturbance of lipid uptake and cholesterol efflux results in the transformation of macrophages into foam cells.

Interleukin-34 (IL-34) has been recently discovered by functional screen of the extracellular proteome. It is expressed broadly in various tissues such as the brain, liver, spleen and heart^9^. IL-34 is involved in varietal chronic autoimmune and inflammatory diseases and its potential as therapeutic target has been developed. Serum concentrations of IL-34 in various inflammatory and autoimmune diseases including obesity, insulin resistance (IR), coronary artery diseases (CAD) and rheumatoid arthritis (RA), lupus nephritis are significantly elevated^10–12^. However, there have been no studies currently on whether IL-34 involves in macrophage lipid metabolism. Overexpression of IL-34, along with elevated CRP serum level in atherosclerosis, suggests a possible link between IL-34 and foam cell formation.

In addition, atherosclerosis has been considered as a chronic inflammatory disease characterized by the recruitment of immunocytes, and a devil of inflammatory cytokines involved in macrophage lipid metabolism^13^. The specific targeting of these processes in experimental models has been shown to withhold the pathologic progression of atherosclerosis. Therapies targeted in inflammatory processes of cardiovascular disease are promising.

In our present study we explored the contribution of IL-34 to foam cell formation and the underlying mechanism for the first time. We confirmed that exogenous administration of IL-34 to BMDMs promotes lipid accumulation. Macrophages activated with IL-34 *in vitro* had an augmented capacity of oxLDL uptake by an enhancement of mRNA and protein expression of CD36 as well as aggravated secretion of pro-inflammatory cytokines and resulted in promoting foam cell formation. In addition, IL-34 showed no significant effect on cholesterol efflux, in consistent with invariant expression of ABCA1, ABCG1 and SR-B. Taken together, our results reveal the contribution of IL-34 to foam cell formation, which is the key initial process of atherosclerosis, provide a new clue to clarify the underlying mechanism of atherosclerosis, and suggest that IL-34 might be a new risk biomarker for atherosclerosis diagnosis. Targeting this new cytokine might be a potential therapeutic target for atherosclerosis-based cardiovascular diseases.

## Materials and Methods

This study was approved by the Chinese PLA General Hospital Ethical Committee. All methods were performed in accordance with the relevant guidelines and regulations.

### Reagents

NF-κB inhibitor Caffeic acid phenethyl ester (CAPE) was purchased from Sigma. ERK1/2 inhibitor PD98059, JNK inhibitor SP600125 and p38 inhibitor SB203580 were purchased from Santa Cruz.

### Mice bone marrow-derived macrophages isolation and treatments

Mice on the C57BL/6 background were obtained from the Chinese Academy of Military Medicine Science Animal Center and housed in specific pathogen-free conditions (SPF). 8 to 12 weeks old littermates were used in this study and all experimental procedures in mice were approved by the Chinese PLA General Hospital Ethical Committee. BMDMs were isolated as previously described^14^. Femur and femur head were separated and bone marrow was flushed out with PBS. The suspended cells were filtered through a 70 μm strainer to remove any impurity. The single cell suspension was then centrifuged and resuspended, plated in RPMI-1640 medium containing 10% fetal bovine serum (FBS), 30% L929 cell-condition medium (LCM, as a supplier of M-CSF) for 6 days to differentiate into macrophages. Fresh medium was replaced every two days. Cells were used by day 7. Identification of BMDMs are shown in Supplementary Fig. 1. For most experiments, differentiated macrophages were pretreated with IL-34 (20 ng/ml or 50 ng/ml) or PBS for 8 hr, subsequently co-incubated with 50 μg/ml oxLDL for another 16 hr in RPMI 1640 with 10% FBS.

### Foam cell formation assay

BMDMs were plated on coverslips in 12-well plates. After treatment as described above, foam cells were determined by Oil Red O staining. Oil Red O (Sigma-Aldrich) working solution was diluted with ddH_2_O in 3:2 homogeneously, standing still for 10 min. BMDMs grown monolayer were incubated with oxLDL in the presence or absence of IL-34 on 12-well plates as indicated above. Macrophages were washed with PBS three times, fixed with 4% formaldehyde (in PBS) for 15 min and rinsed in PBS. Then cells were stained for 30 min with filtered Oil Red O working solution at room temperature and rinsed with PBS, 60% isopropanol (in PBS) three times. Cells were then stained with hematoxylin for 5 sec and rinsed in PBS. Pictures were taken by optical microscope.

### Cellular cholesterol and cholesteryl ester measurements

Cellular total cholesterol, free cholesterol and cholesteryl ester in BMDMs treated with IL-34 or oxLDL as described previously were analyzed using the Cholesterol/CE Quantitation Kit II (Biovision) in compliance with the manufacturer’s instruction. The concentration of cellular-protein was calculated using Pierce BCA Protein Assay Kit (Thermo Scientific). Three independent assays were performed.

### Analysis of Dil-oxLDL uptake

For uptake study, fluorescence-labelled oxidative LDL (Dil-oxLDL, Unionbiol, Beijing, China) was used. After pretreated with IL-34 or PBS, adherent cells were co-incubated with Dil-oxLDL (50 μg/ml) in 37 °C for 4 hr. For functional neutralizing assay, cells were pretreated with 10 μg/ml anti-CD36 antibody or goat IgG (both R&D Systems) or SB203580 (10 μM) for 1 hr, subsequently co-cultured with Dil-oxLDL for 4 hr at 37 °C. Fluorescence intensity was detected using fluorescence microscope and calculated using Image Pro Plus software. Three independent experiments were performed. Alternatively, detection of Dil-OxLDL uptake by flow cytometry. Cells were washed with cold PBS for 2 times before any staining and then cells were detached from the plate, and cell surface markers were stained on ice for 30 min. After two times of wash by FACS washing buffer (2% FBS in PBS), samples were analyzed by FACS. Data were further quantified by software FlowJo.

### Cholesterol efflux analysis

Analysis of cholesterol efflux was prepared as previous described^15^. BMDMs were differentiated into foam cells by co-cultured with oxLDL and 2 µCi/ml of [^3^H] cholesterol (Perkin Elmer) for 24 hr. Cells were washed twice in PBS, and fresh medium containing either 2 mg/ml BSA (Sigma-Aldrich), 10 ug/ml ApoA-I (Sigma-Aldrich) or HDL (Sigma-Aldrich) was replaced, subjected to efflux for 12 hr at 37 °C. The medium was collected and centrifuged at 12000 rpm for 10 min to remove rudimental cell fragmentations. Cells were solubilized in water by one hour swing. ^3^H radioactivity in medium and cells were detected using liquid scintillation counting respectively. Cholesterol efflux was determined as the percentage of radioactivity in the medium relative to the total radioactivity (cells + medium).

### Measurements of inflammatory cytokines secretion

Concentrations of IL-6, TNF-α and IL-1β were measured in the supernatants obtained from the control and ox-LDL stimulated BMDMs in the presence or absence of IL-34 pretreatment by using ELISA Kits (all from BD Biosciences) according to the manufacturer’s instruction.

### Western blot analysis

BMDMs were seeded onto 6 well plates. After treatment as described above, cells were washed with PBS and lysed in HEPES buffer (20 mM HEPES PH7.2, 50 mM NaCl, 0.5% TritonX-100, 1 mM NaF, 1 mM DTT, 5 mM EDTA) containing protease inhibitors on ice. The lysates were collected and boiled for 20 min and separated on 10% SDS-PAGE, then transferred to nitrocellulose membranes, blocked with skim milk, incubated with primary antibodies and specific secondary antibodies, and specific proteins were detected using chemiluminescence method and the bands intensities were scanned and calculated by densitometry (NIH ImageJ software). Relative amounts of each protein were normalized by calculated. These following antibodies were used. Anti-SR-B, ABCG1 and β-actin antibodies were purchased from Santa cruz. Anti-CD36, SR-A, LOX-1 antibodies were purchased from R&D system, and anti-ABCA1 antibody was obtained from Abcam. Anti-p-p38, p-p65, p-IκB, p-erk, p-JNK antibodies were from Cell Signaling Technology.

### Quantitative Real-time PCR

Total RNA was extracted using the Trizol reagent (Sigma-Aldrich), and then reverse transcribed into cDNA using the Rever Tra Ace qPCR RT Kit (ToYoBo). Real-time quantitative PCR analysis was determined using KAPA SYBR FAST qPCR Kit Master Mix (KAPABIO SYSTEM). Primers used in this study were seen in Supplementary Table 1. Threshold cycle number of each gene was determined, and β-actin was used as the internal control to quantify the relative expression.

### Statistical analysis

All results were expressed as means ± SD with the indicated number of independent experiment. A two-tailed Student’s *t* test was used to calculate the significance differences between two groups. Comparison of more than two groups was made using a one-way analysis of variance (ANOVA) followed by Dunnett’s *post hoc* test. All statistical analyses were performed with GraphPad Prism 6 and SPSS 22.0 software. Significance was accepted at the level of *P* < 0.05.

## Results

### IL-34 enhanced macrophage cholesterol accumulation and promoted foam cell formation

**Figure 1:**
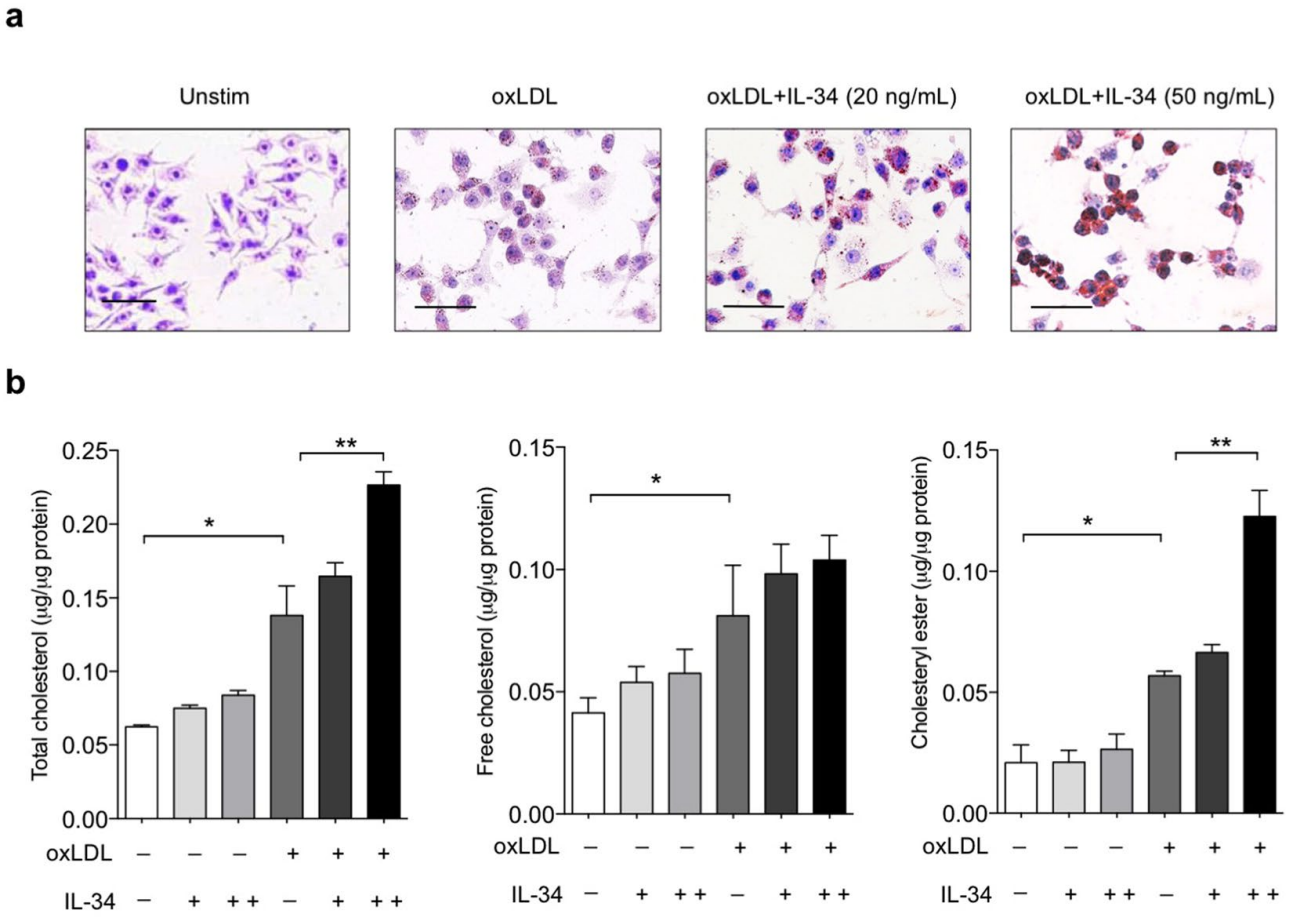
Effect of IL-34 on foam cells formation in BMDMs. (**a**) IL-34 promoted foam cell formation. Macrophages were incubated with either medium alone, oxLDL (50 μg/ml), oxLDL (50 μg/ml) + IL-34 (20 ng/ml) or oxLDL (50 μg/ml) + IL-34 (50 ng/ml) for 24 hr. Oil red O staining was performed. Representative micrographs were showed. (**b**) IL-34 enhanced total cholesterol and cholesteryl ester accumulation. Macrophages were treated with oxLDL (50 μg/ml) in the presence or absence of IL-34 20 ng/ml, 50 ng/ml respectively for 24 hr. Total cholesterol, free cholesterol and cholesteryl ester contents were detected. Data represent mean ± SD of *n* = 3 biologically independent experiments. *P < 0.05, **P < 0.01.

We pretreated BMDMs with IL-34 and then loaded the cells with oxLDL for 24 h. Oil Red O staining clearly revealed that IL-34 treatment significantly promoted a noticeable increase of accumulation of intracellular lipid droplets in a dose dependent manner (Fig. 1a). Cellular content of free cholesterol and cholesteryl ester were detected by direct chemical measurement (Fig. 1b). Without oxLDL, cholesterol and cholesteryl ester content of IL-34-treated macrophages were indistinguishable from that of untreated cells. However, after 16 hr treatment of oxLDL, IL-34 significantly promoted the accumulation of cholesteryl ester by about 2-fold compared with the untreated controls. No significant difference was found in the content of intracellular free cholesterol between IL-34-treated and untreated cells (Fig. 1b). Thus IL-34 enhanced the cholesteryl ester accumulation and promoted macrophage foam cell formation.

### IL-34 enhanced Dil-oxLDL uptake through increasing the expression of scavenger receptor CD36

**Figure 2:**
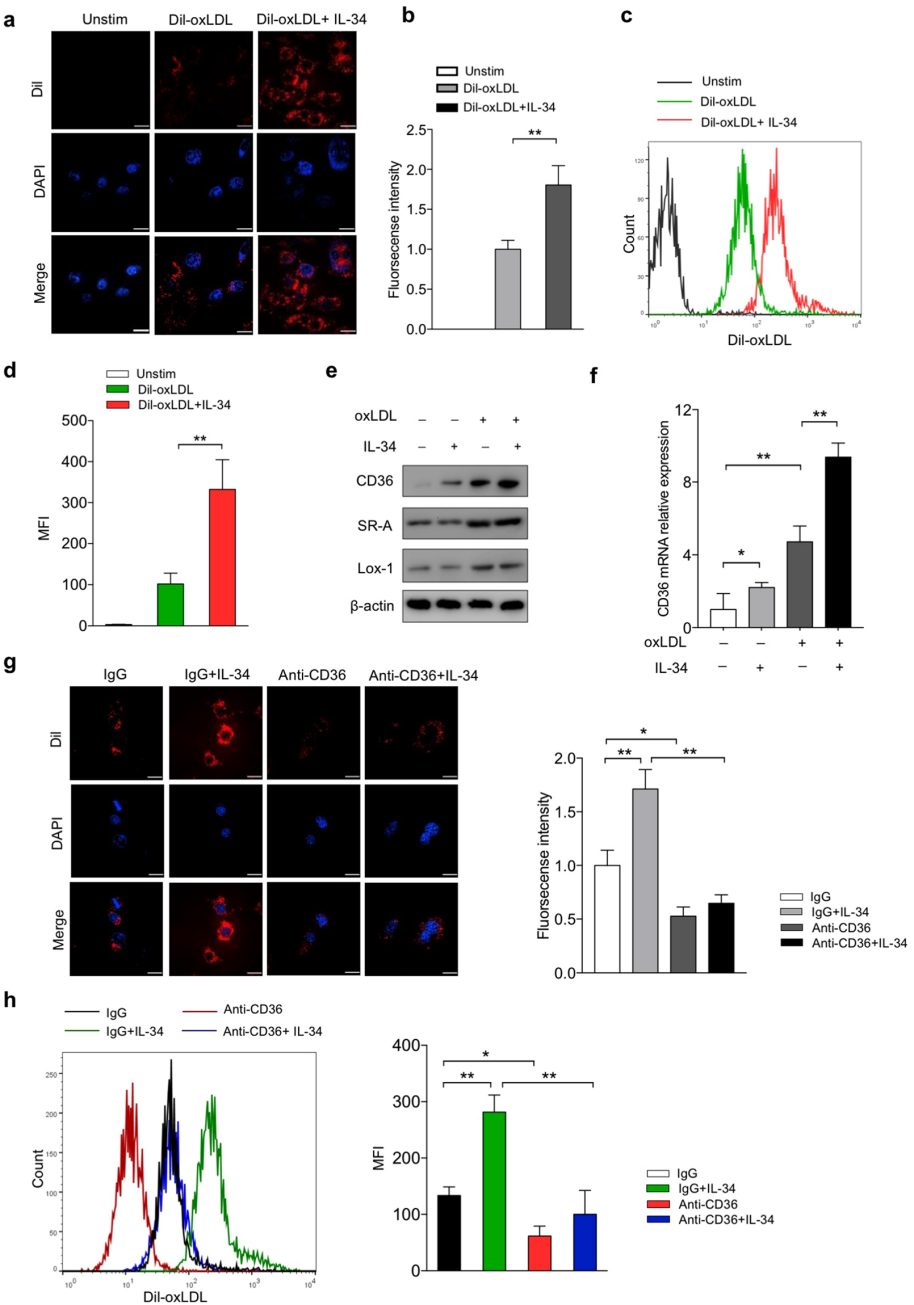
IL-34 increased Dil-oxLDL uptake in BMDMs through up-regulation of expression of CD36. BMDMs were treated either with medium alone (control), with Dil-oxLDL (50 μg/ml), or with Dil-oxLDL (50 μg/ml) + IL-34 (50 ng/ml) for 4 hr at 37 °C followed by confocal microscopy (**a**,**b**) or flow cytometry (**c**,**d**) to determine the uptake of Dil-oxLDL. (**e**) Expression of scavenger receptors CD36, SR-A and Lox-1 protein in BMDMs by western blot. (**f**) Analysis of mRNA levels of CD36 was carried out by real-time quantitative PCR. For a neutralizing assay, BMDMs were pre-incubated with 10 μg/ml anti-CD36 antibody or normal goat IgG for 1 hr followed by treated with either Dil-oxLDL or Dil-oxLDL + IL-34. Dil-oxLDL uptake was determined by confocal microscopy (**g**,**h**) or flow cytometry (**i**,**j**). Data represent mean ± SD of *n* = 3 biologically independent experiments. *P < 0.05, **P < 0.01. Unprocessed original scans of blots are shown in Supplementary Fig. 3.

Since the influx and efflux of the oxLDL together determine the formation of the foam cells, the increase of foam cell formation may be due to the increase of oxLDL uptake or the deficiency of degraded oxLDL efflux or both. To investigate the contribution of IL-34 to oxLDL uptake by macrophages, we used confocal microscopy and flow cytometry to confirm the role of IL-34 in lipid uptake. Dil-labeled oxLDL uptake assay was performed. Cells were washed with ice-cold acid buffer to avoid potential contamination of the cell surface with “sticky” oxLDL before detection. We found that the oxLDL uptake by macrophages was significantly increased in IL-34-treated cells than that in untreated cells (Fig. 2a–d).

Next, to determine whether IL-34 promotes Dil-oxLDL uptake through up-regulation of the expression of scavenger receptors, we examined the effects of IL-34 on the expression of CD36, SR-A and Lox-1, major scavenger receptors that mediate the uptake of modified LDL, such as oxLDL and acLDL. As shown in Fig. 2e and Supplementary Fig. 2a, treatment with IL-34 markedly increased the protein expression of CD36, but no such increase of SR-A and Lox-1 expression was observed. To further study whether IL-34 affects transcription of scavenger receptor genes, we also investigated the mRNA levels of these proteins by RT-PCR and revealed significantly increase of CD36 mRNA with no big changes on SR-A and Lox-1 mRNA levels (Fig. 2f and Supplementary Fig. 2b). To further examine the importance of CD36 in modified LDL uptake in response to IL-34, antibody-dependent blocking assay was performed. As expected, re-incubation with anti-CD36 antibody decreased Dil-oxLDL uptake compared to isotype control, and no marked difference was seen between IL-34-treated macrophages and untreated cells (Fig. 2g,h). Taken together, our results indicate that IL-34 enhanced oxLDL uptake in BMDMs, at least in part, through the up-regulation of CD36 expression.

### IL-34 had no significant effect on cholesterol efflux

**Figure 3:**
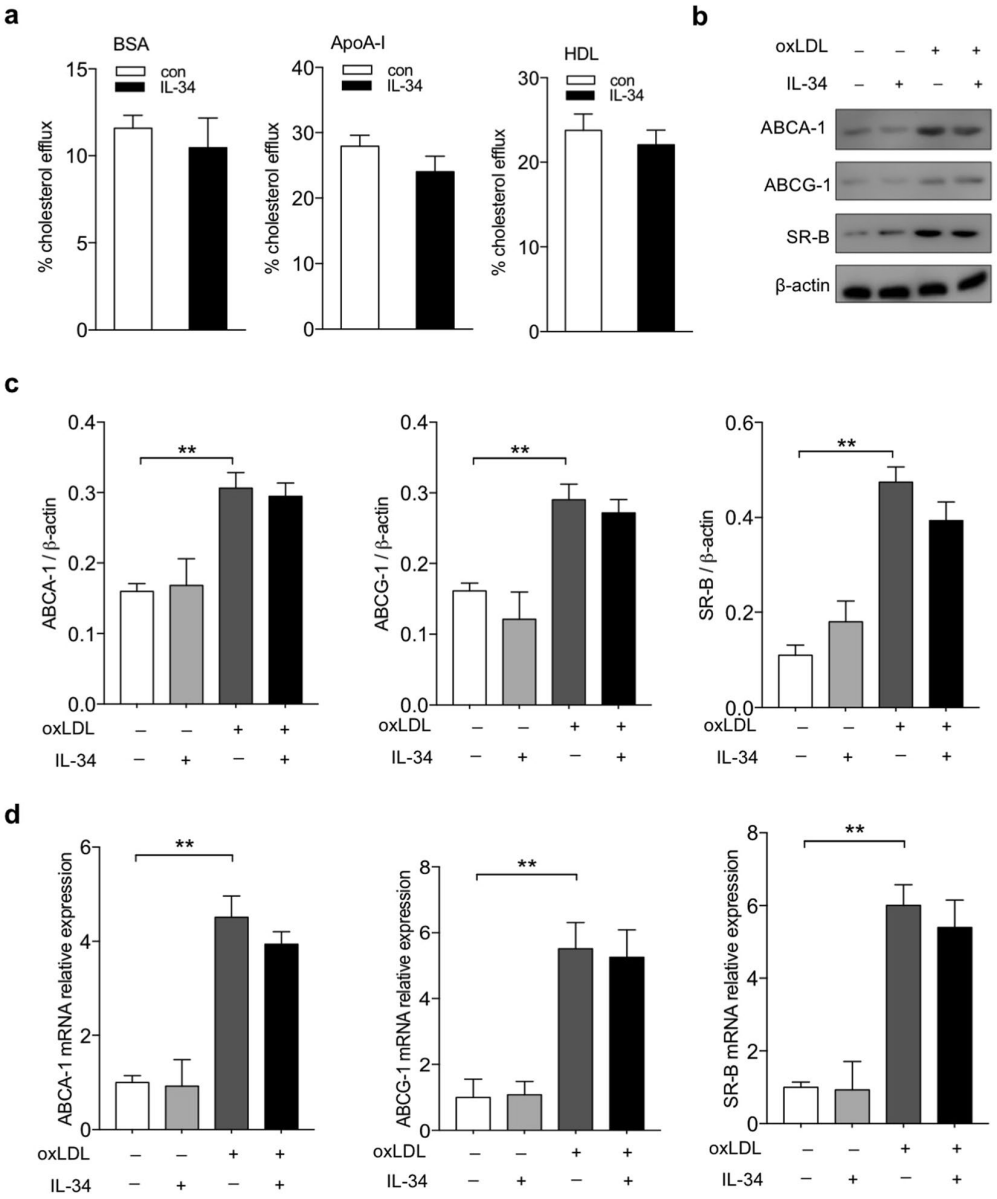
IL-34 had no significant influence on cholesterol efflux in BMDMs. (**a**) Non–dependent, ApoA-I-dependent or HDL-dependent cholesterol efflux was measured by incubating [^3^H] cholesterol-labeled macrophages with (+apoA-I, +HDL) or without (−apoA-I,−HDL) for 12 hr. Radioactivity in the medium was determined as a percentage of total radioactivity in the cells and medium. (**b**) Expression of ABCA1, ABCG1 and SR-B was examined by Western blot. (**c**) Quantitative data were presented of three independent experiments. (**d**) Quantitative real-time PCR was used to measure ABCA1, ABCG1 and SR-B mRNA levels. Data represent mean ± SD of *n* = 3 biologically independent experiments. *P < 0.05, **P < 0.01. Unprocessed original scans of blots are shown in Supplementary Fig. 3.

The unbalance of lipid-uptake and cholesterol efflux leads to the overloaded lipid of macrophages and foam cells formation. We next investigated whether IL-34 plays a role in macrophage cholesterol efflux. ^3^H-labeled cholesterol tracer was used to analyze the efflux to lipid-poor ApoA-I or HDL. IL-34 showed no apparent decrease in cholesterol efflux to both ApoA-I and HDL compared with the control group (Fig. 3a). Expression of ABC transporters related to oxLDL efflux, including ABCA1, ABCG1, and SR-B were investigated. OxLDL significantly increased the natural expression of ABCA1, ABCG1 and SR-B. Western blot results showed that no significant changes were found for the natural expression or induced expression by oxLDL of ABCA1, ABCG1 and SR-B after IL-34 treatment (Fig. 3b,c). In consistent with the results of protein expression, mRNA expression of ABCA1, ABCG1 and SR-B shows no difference response to IL-34 (Fig. 3d). These experimental results suggest that IL-34 has no effect on cholesterol efflux in BMDMs.

### IL-34 up-regulated CD36 expression partially via p38 MAPK pathway

**Figure 4:**
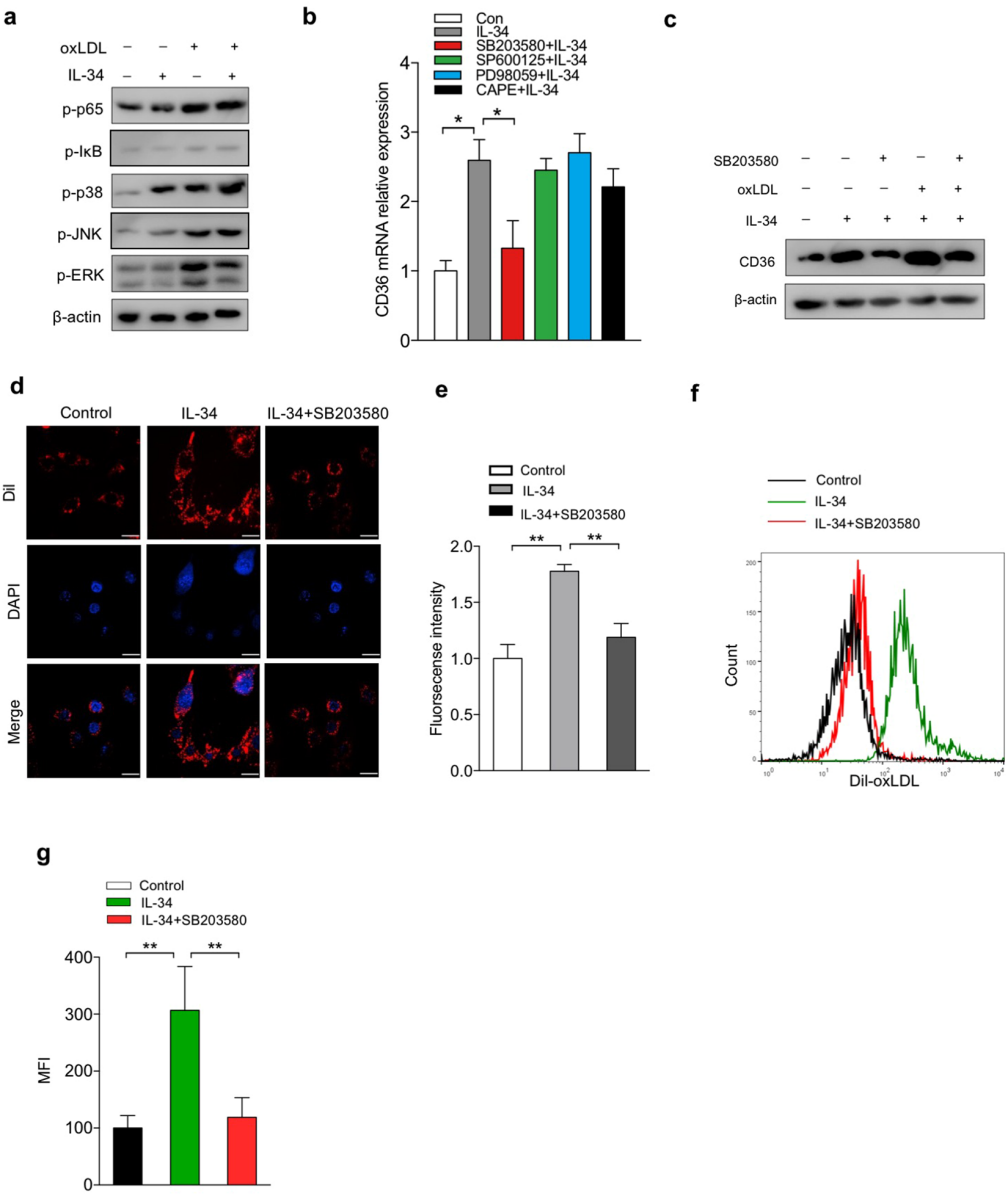
IL-34 up-regulated CD36 expression and promoted foam cell formation partially via p38 MAPK pathway. (**a**) Effects of IL-34 on the protein expression of phosphorylation of NF-κB p65, IκB, p38 MARK, JNK and ERK were assessed by western blot. Macrophages were pretreated with CAPE (10 μM), SP600125 (10 μM), PD98059 (10 μM), SB203580 (10 μM) respectively for 1 hr, following co-incubated with IL-34 for 24 hr. CD36 mRNA transcripts (**b**) and protein levels (**c**) were analyzed. Macrophages were pretreated with or without SB203580 for 1 hr, and followed by treated with either Dil-oxLDL or Dil-oxLDL + IL-34 as indicated. Fluorescence confocal microscopy (**d**,**e**) or flow cytometry (**f**,**g**) were carried out to determine total uptake of Dil-oxLDL in each group. Data represent mean ± SD of *n* = 3 biologically independent experiments. *P < 0.05, **P < 0.01. Unprocessed original scans of blots are shown in Supplementary Fig. 3.

Mitogen-activated protein kinases (MAPK) contain three major members: p38 MAP kinase, extracellular signal-regulated kinase (ERK) and c-Jun N-terminal kinase (JNK). Considering the importance of MAPK and nuclear factor (NF)-κB in regulating CD36 scavenger expression and many inflammatory genes, we analyzed the protein expression of phosphorylation of NF-κB p65, IκB and p38, JNK, ERK, to further clarity mechanisms through which IL-34 enhances CD36 expression and leads to lipids accumulation in macrophages. OxLDL definitely induced phosphorylation of p65, p-IκB and p38, JNK, ERK compared with the control group (Fig. 4a). After treatment with IL-34, we observed a higher phosphorylation of p38, but no big difference of phosphorylation of p65, p-IκB, JNK and ERK were found between IL-34-pretreatment macrophages and untreated cells (Fig. 4a). To further elucidate whether IL-34 results in changes in CD36 expression by influencing transcriptional levels, we studied CD36 mRNA level after pretreatment with p38 inhibitor SB203580, JNK inhibitor SP600125, ERK inhibitor PD98059 and NF-κB inhibitor CAPE respectively. The results showed that it was the p38 inhibitor that markedly decreased the expression of CD36 induced by IL-34 (Fig. 4b). Consistently, CD36 protein expression induced by IL-34 was evidently suppressed with SB203580 pretreatment (Fig. 4c). The IL-34-induced Dil-oxLDL uptake was suppressed by pretreatment with SB203580 (Fig. 4d–g), which was consistent with the expression of CD36. These results implied that p38 pathway may involve in the up-regulation of CD36 and IL-34-induced enhancement of foam cell formation.

### IL-34 promoted oxLDL-induced inflammatory molecules in BMDMs

**Figure 5:**
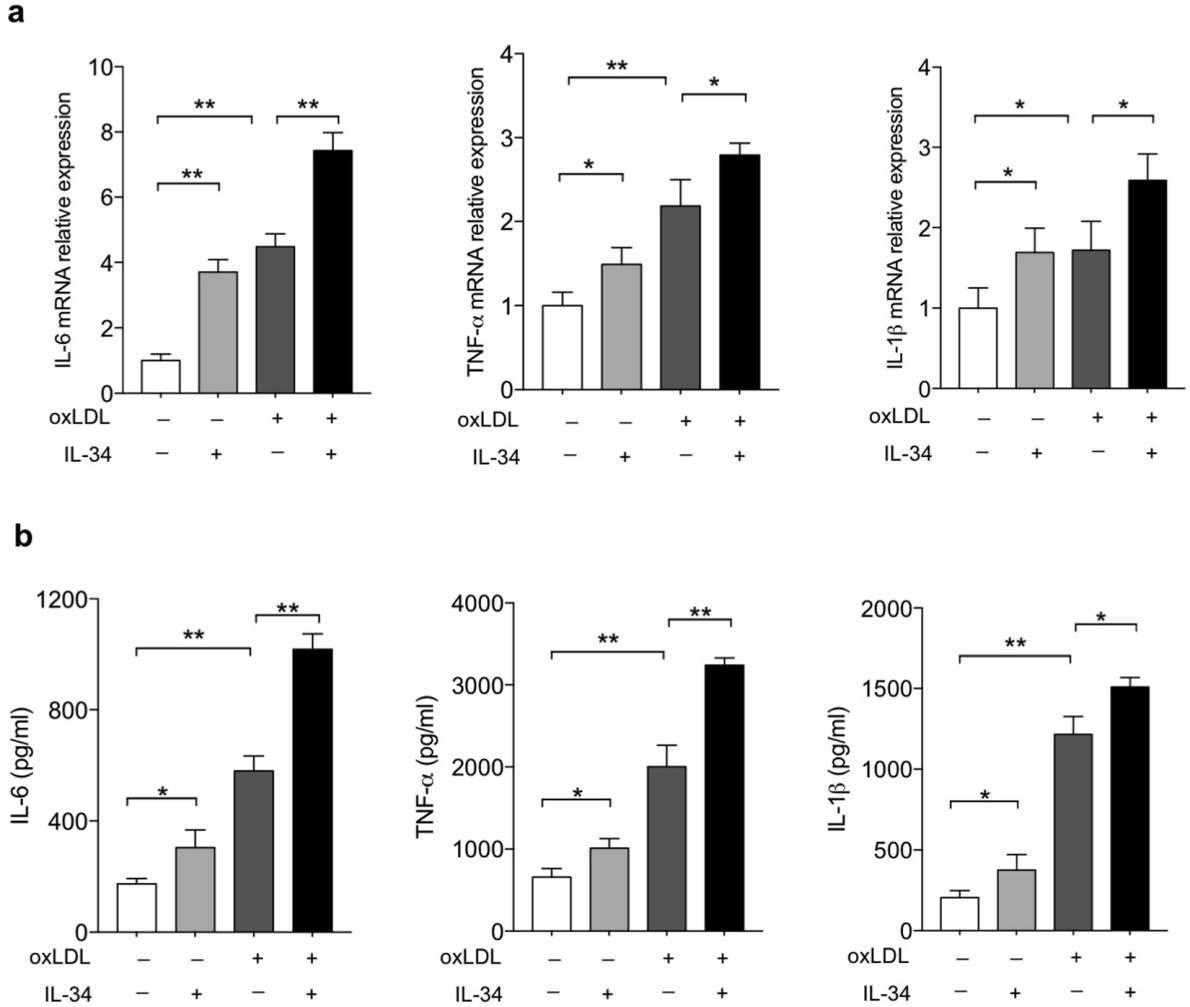
IL-34 enhanced oxLDL-induced inflammation in BMDMs. (**a**) BMDMs were pre-incubated with IL-34 (50 ng/ml) or oxLDL (50 μg/ml) for the indicated times. IL-6, TNF-α and IL-1β mRNA expression were quantified by RT-PCR. (**b**) BMDMs were pre-incubated with IL-34 (50 ng/ml) or oxLDL (50 μg/ml) for the indicated times and then the cells were activated by lipopolysaccharide (LPS) for an additional 3 hr. Supernatants were collected after stimulation and IL-6, TNF-α and IL-1β concentrations were measured by ELISA. Data represent mean ± SD of *n* = 3 biologically independent experiments. *P < 0.05, **P < 0.01.

Inflammation is a hallmark of atherosclerosis. To assess the role of IL-34 on pro-inflammatory cytokines in macrophages, the mRNA expression of IL-6, TNF-α and IL-1β in macrophages and the concentration of these cytokines in the culture medium were detected. Stimulated by IL-34, the mRNA expression of IL-6, TNF-α and IL-1β were significantly increased, which were enhanced by oxLDL (Fig. 5a). To confirm the results obtained from RT-PCR experiments, we measured cytokine secretion to the medium after cell activation by lipopolysaccharide (LPS). In line with the RT-PCR experiments, the cytokine concentration of IL-6, TNF-α and IL-1β in the culture medium markedly increased after exposure to IL-34, when compared to the untreated control group (Fig. 5b). These results suggest that IL-34, despite its role in enhancing cholesterol esters accumulation and foam cell formation, had a dramatic up-regulation effect on the expression of pro-inflammatory molecules in BMDMs.

## Discussion

Foam cell involves in the formation of fatty streak and stability of atheromatous plaques, playing a crucial role in the pathologic processes of atherosclerosis. The relationship of secretion and regulation of interleukins and atherosclerosis had been widely studied, and may provide novel direction to outstanding questions of atherosclerosis. A few studies have reported that level of IL-34 was highly elevated in tissues, blood or synovial fluid from patients with RA, IBD and sjogren syndrome (SS)^16–18^. Thus, depending on this, inhibition of IL-34 pathway could be a beneficial anti-inflammatory therapeutic method. Furthermore, IL-34 has been considered being associated with coronary artery disease, obesity and chronic inflammation^10,12^. This suggests that the newly identified IL-34 cytokine mediates distinct biologic activities and a potential link between atherosclerosis. However, its role in foam cell formation still remains unknown. The present study expands upon our exploration of IL-34 in regulating macrophage foam cell formation and elucidates the underlying signaling pathway. Our major finding in this study is that IL-34 enhances macrophage oxLDL uptake and promotes atherogenic lipid oxLDL-induced foam cell formation *in vitro* for the first time. Mechanistically, this phonotype is on account of the enhanced mRNA and protein expression of scavenger receptor CD36 and increased secretions of inflammatory cytokines, thereby facilitating oxLDL uptake and lipid deposition in macrophages and enhanced oxLDL-uptake could be blocked by the inhibition of p-38 MAPK signaling pathway.

Lipid uptake and cholesterol efflux are co-responsible for foam cell formation. Therefore, we firstly detected the influence of IL-34 on macrophage lipid uptake and scavenger receptors expression, including CD36, SR-A and Lox-1^19–21^. Our study shows that although IL-34 does not significantly increase SR-A and Lox-1 protein and mRNA expression induced by oxLDL compared with the control group, expression of CD36 is markedly increased. Scavenger receptors play a key role in foam cell formation. Numerous scavenger receptor family members involves in foam cell formation^22^, among them, CD36 and SR-A account for 75–90% of modified LDL uptake by macrophage *in vitro*^4,23^. CD36 belongs to scavenger receptor class B family, binds multiple ligands including oxLDL, thrombospondin-1, cell-derived microparticles and apoptotic cells^24^. The internalization of oxLDL through CD36 provokes the activation of the transcription factor NF-κB^25,26^, and the productions of inflammatory cytokines such as IL-6, TNF-α and IL-1β are raised, promoting the atherosclerotic inflammatory process^23,27,28^. According to this, we analyzed the expression of pro-inflammatory cytokines. The excitatory effect of IL-34 on the productions of IL-6, TNF-α and IL-1β, which are typical pro-inflammatory cytokines, may also contribute to its enhancement on foam cell formation. Consequently, we speculate that IL-34 promotes lipid accumulation in macrophages may be, at least in part, attributed to its augmentation of CD36 expression and secretions of pro-inflammatory cytokines.

We also tested whether IL-34 has a primary defect in cholesterol efflux. Addition of IL-34 showed no reduction of cholesterol efflux by assay using [^3^H] cholesterol tracer, and no noticeable effect on the expression of major mediators of cholesterol efflux ABCA1, ABCG-1 and SR-B were seen. In conclusion, data presented in this study demonstrate that IL-34 promotes foam cell formation by mainly regulating the expression of genes for oxLDL uptake, pro-inflammatory cytokines but not cholesterol efflux.

MAPK involves in different biological processes including apoptosis or survival, proliferation, differentiation, inflammation, autophagy and also plays a considerable role in the pathogenesis of atherosclerosis^29,30^. Activation of p38 has been reported to regulate oxLDL-induced CD36 expression and foam cell formation^31–33^. We found in our study that IL-34 induces p38 phosphorylation even without oxLDL stimulation, suggesting that p38 plays an important role in the enhancement of IL-34-induced CD36 expression. Using specific inhibitor of p38, we found that protein and mRNA expression of CD36 induced by IL-34 is decreased. Notably, with the presentation of p38 inhibitor, increase of oxLDL uptake induced by IL-34 is blocked, indicating that IL-34 probably induces CD36 mRNA expression via p38 signaling pathway.

Atherosclerosis has been the key cause of death worldwide. However, the underlying molecular mechanism still needs further research. This report, to our knowledge, is the first study providing evidence for the role of IL-34 in foam cell formation. More studies are needed to understand the contribution of IL-34 to foam cell formation and the pathologic processes of atherosclerosis, and to identify new opportunities for targeting this new cytokine for clinical benefit.

## Electronic supplementary material

Supplementary information

## Data Availability

The authors declare that all the relevant data supporting the findings of this study are available within the article and its Supplementary Information files, or from the corresponding author on reasonable request.
