# Supplementary material for: IL-34 promotes foam cell formation by enhancing CD36 expression through p38 MAPK pathway
Source: Sci Rep. 2018 Nov 26;8:17347. doi: 10.1038/s41598-018-35485-2 (PMC6255782; doi:10.1038/s41598-018-35485-2)
Supplement: Supplementary file 1 — Supplementary information [file 41598_2018_35485_MOESM1_ESM.pdf]

# IL-34 promotes foam cell formation by enhancing CD36 expression through p38 MAPK pathway

Qingyan Liu, Jiao Fan, Jing Bai, Liang Peng, Tao Zhang, Lei Deng, Gaokun Wang,

Yu Zhao, Jingguo Nong, Minghua Zhang, Yu Wang

## Supplementary Figures

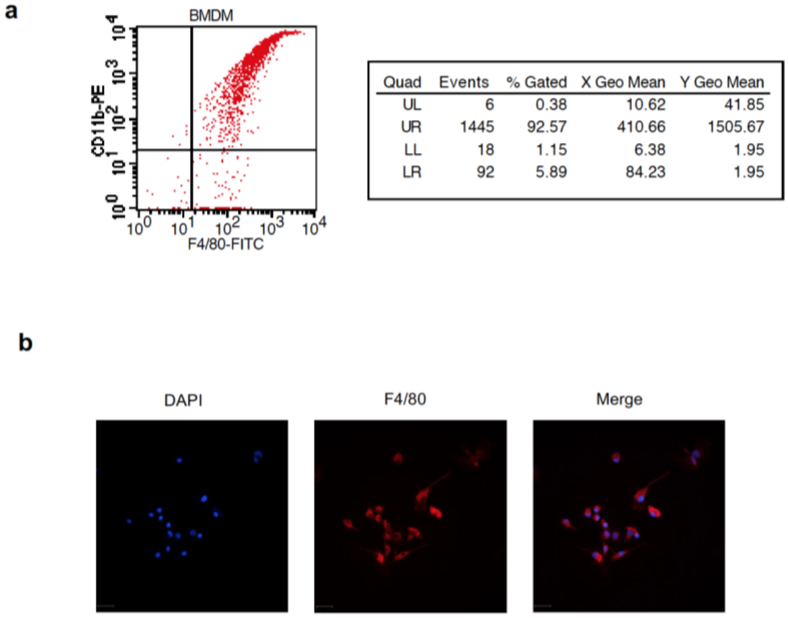


| Quad | Events | % Gated | X Geo Mean | Y Geo Mean |
|---|---|---|---|---|
| UL | 6 | 0.38 | 10.62 | 41.85 |
| UR | 1445 | 92.57 | 410.66 | 1505.67 |
| LL | 18 | 1.15 | 6.38 | 1.95 |
| LR | 92 | 5.89 | 84.23 | 1.95 |

**Supplementary Figure 1.** Identification of BMDMs. (**a**) Flow cytometry was carried out to determine the purity of BMDMs. Specific markers CD11b and F4/80 were used. (**b**) Immunofluorescence staining of F4/80 to identify the BMDMs.

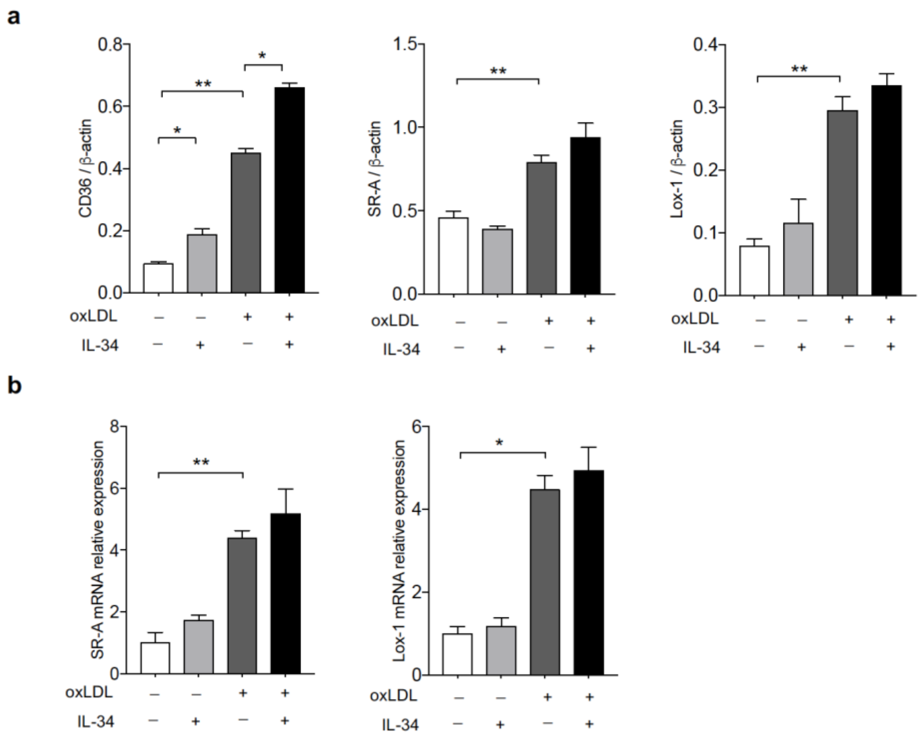


**Supplementary Figure 2.** IL-34 enhanced the expression of scavenger receptor CD36 in BMDMs. (**a**) Quantitative data of the protein expression of CD36, SR-A and LOX-1 for Fig. 2e were presented of three independent experiments. (**b**) Analysis of mRNA levels of SR-A and LOX-1 was carried out by real-time quantitative PCR. Data represent mean ± SD of $n = 3$ biologically independent experiments. *$P < 0.05$, **$P < 0.01$.

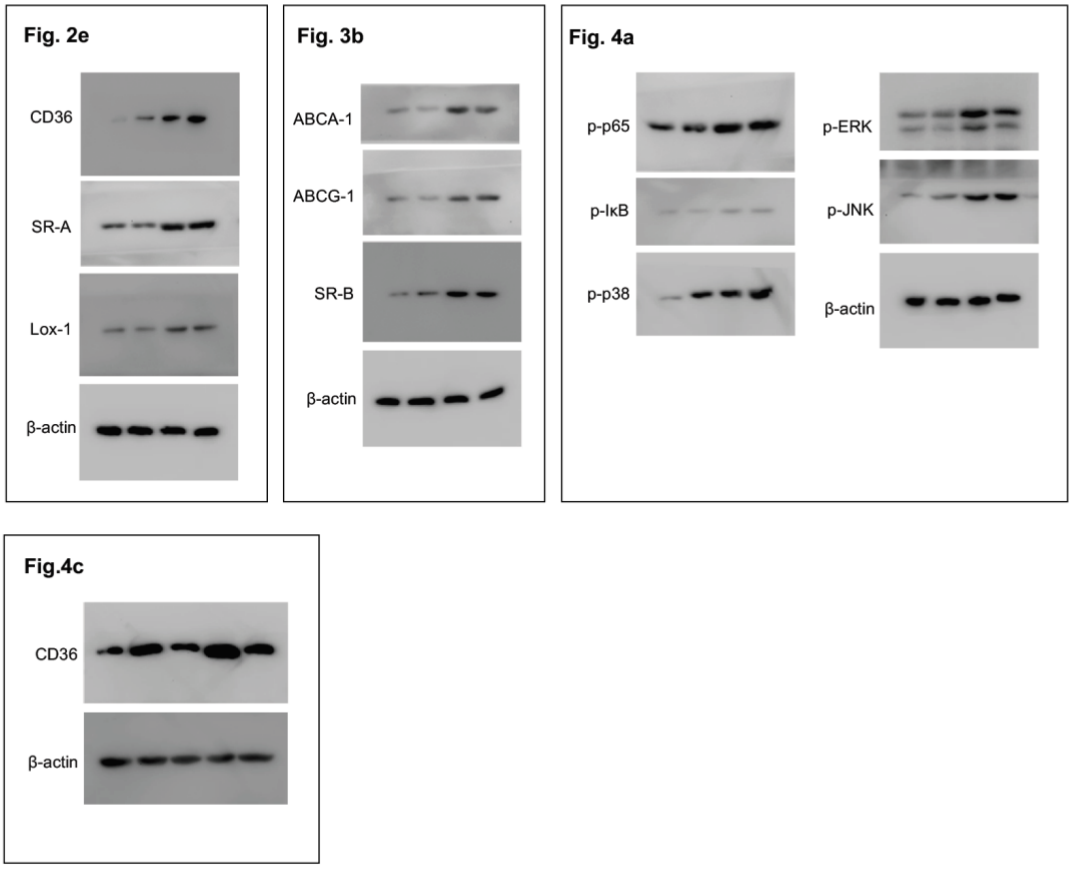


**Supplementary Figure 3.** Uncropped images of Fig. 2e, 3b, 4a and 4c.

## Supplementary Tables

**Supplementary Table 1.** Sequence of Primers used in the present study.

| **RT-PCR** | ***Forward*** | ***Reverse*** |
|---|---|---|
| CD36 | AGGAATTTGTCCTATTGGGAAAGTT | CCGCAGTACCCGAGACTTCT |
| SR-A | GGGAACACTCACAGACACTGAAA | GGGTTGATCCGCCTACACTC |
| LOX-1 | CAAGATGAAGCCTGCGAATGA | ACCTGGCGTAATTGTGTCCAC |
| ABCA1 | ATAGCAGGCTCCAACCCTGAC | GGTACTGAAGCATGTTTCGATGTT |
| ABCG1 | GGGATCAGAACAGTCGCCTG | CGAGGTCTCTCTTATAGTCAGCGTC |
| SR-B | TTTGGAGTGGTAGTAAAAAGGGC | TGACATCAGGGACTCAGAGTAG |
| IL-1β | GCCCATCCTCTGTGACTCA | AGGCCACAGGTATTTTGTCG |
| IL-6 | TAGTCCTTCCTACCCCAATTTCC | TTGGTCCTTAGCCACTCCTTC |
| TNF-α | CCCTCACACTCAGATCATCTTCT | TGCTACGACGTGGGCTACAG |
| β-actin | TGCTGTCCCTGTATGCCTCTG | AGGGAGAGCGTAGCCCTCAT |
